# Loss of interleukin-12 modifies the pro-inflammatory response but does not prevent duct obstruction in experimental biliary atresia

**DOI:** 10.1186/1471-230X-6-14

**Published:** 2006-04-19

**Authors:** Sujit Kumar Mohanty, Pranavkumar Shivakumar, Gregg Sabla, Jorge A Bezerra

**Affiliations:** 1 Division of Gastroenterology, Hepatology and Nutrition, Cincinnati Children's Hospital Medical Center, and Department of Pediatrics of the University of Cincinnati, Cincinnati, OH, USA

## Abstract

**Background:**

Livers of infants with biliary atresia and of neonatal mice infected with rotavirus (RRV) have increased expression of interferon-gamma (IFNγ) and interleukin (IL)-12. While the expression of IFNγ regulates the obstruction of extrahepatic bile ducts by lymphocytes, the role of IL-12 in the pathogenesis of biliary obstruction is unknown. Based on the role of IL-12 as a key proinflammatory cytokine, we hypothesized that loss of IL-12 prevents the obstruction of extrahepatic bile ducts.

**Methods:**

IL12-knockout (IL-12KO) and wild type mice were injected with RRV or saline at day 1 of age and monitored for the development of symptoms. The cellular and molecular phenotypes were determined at days 3, 7, and 14 by real-time PCR and flow cytometry.

**Results:**

RRV infection of IL-12KO mice resulted in growth failure, jaundice/acholic stools, and decreased survival similar to wild-type mice. IL-12KO mice had a remarkable neutrophil-rich portal inflammation and epithelial sloughing of extrahepatic bile ducts. Loss of IL-12 decreased but did not abolish the hepatic expression of IFNγ, displayed a remarkable increase in expression of TNFα, IFNα, IFNβ and decreased expression of IL-4 and IL-5.

**Conclusion:**

Loss of IL-12 did not modify the progression of bile duct obstruction in experimental biliary atresia. However, the inflammatory response was predominantly neutrophil-based and displayed a Th1 response in the absence of IL-12.

## Background

Elucidation of the molecular mechanisms regulating injury and obstruction of extrahepatic bile ducts is critical to understand the pathogenesis of biliary atresia, the most common cause of pathological jaundice in infants. In biliary atresia, the extrahepatic bile ducts undergo progressive inflammatory and fibrosing obstruction, which disrupts the lumenal continuity between the biliary tree and the duodenum [1-3]. Although the etiology of the disease is undefined, the pathogenesis involves the interaction of environmental and genetic factors that produce an injury targeting the biliary tree. While the identification of environmental agents in affected patients has varied among different populations, analyses of liver samples obtained from patients at different phases of disease support an important role for inflammatory cells in tissue injury [3]. For example, CD4+ and CD8+ lymphocytes, NK cells and activated macrophages infiltrate the hepatic environment, populating primarily the portal system in diseased tissues, in contrast to the lobular staining found in liver samples from patients with other forms of neonatal cholestasis [4-8]. We recently investigated the transcriptional program in livers of patients with biliary atresia and found a unique footprint containing a significant number of genes related to a proinflammatory activation of lymphocytes at the time of diagnosis, with an increased expression of interferon-gamma (IFNγ) [9].

To directly investigate whether IFNγ played a key role in pathogenesis of biliary injury, we first determined the functional commitment of lymphocytes in the hepatobiliary system in a mouse model of rotavirus-induced biliary atresia [9]. In these mice, inoculation of *rhesus *rotavirus type A (RRV) soon after birth triggered a hepatobiliary inflammation by CD4+ and CD8+ lymphocytes and an overproduction of IFNγ. Then, we applied the same model of rotavirus infection to mice deficient in IFNγ due to a mutation in the *IFNγ *gene. We found that loss of IFNγ in mice prevented the obstruction of the extrahepatic bile ducts by inflammatory cells and maintained biliary continuity between the liver and duodenum [9]. Notably, loss of IFNγ attenuated but did not abolish the hepatic infiltration of CD4+ and CD8+ lymphocytes and allowed for an increase in interleukin (IL)-12 after RRV challenge. Based on these data, on the synergistic role of IL-12 in the regulation of the proinflammatory commitment of lymphocytes, and on the increase in IL-12 expression in livers of children with biliary atresia [7], we hypothesized that loss of IL-12 prevents duct obstruction in experimental biliary atresia.

## Methods

### Mouse model of biliary atresia

Wild-type (WT) Balb/c mice were procured from Charles River Laboratories Inc (Wilmington, MA, USA) and Balb/c mice deficient in IL-12 due to the targeted disruption of the *IL-12p40 *gene (IL-12KO) were procured from the NIH-Taconic consortium. They were maintained in a specific pathogen-free vivarium and housed in a room with a 12-hour dark-light cycle. Within 24 hours of birth, mice were injected with 0.9% saline solution (controls) or 1.5 × 10^6 ^fluorescence forming units (ffu) of RRV intraperitoneally, as described previously [9-11]. Infected mice that died within the first 2 days were excluded from further analysis because early death is associated with complications of injection, such as intra-abdominal bleeding. All mice were weighed daily, examined for the development of icterus of the skin not covered with fur and for the appearance of acholic stools, and sacrificed at 3, 7, and 14 days after saline or RRV challenge. The number of mice used in each experiment and each time point is presented in the *Results *section or in the tables/figure legends. The Institutional Animal Care and Use Committee (IACUC) of the Cincinnati Children's Hospital Research Foundation (Ohio, USA) approved all animal protocols.

### Histopathology and gene expression

Livers and extrahepatic bile ducts were harvested from neonatal mice at 3, 7 and 14 days following saline or RRV using a dissecting microscope. Liver samples and the extrahepatic bile ducts were fixed with formalin, paraffin-embedded, sectioned with microtome (5-μm thickness), and stained with H&E for microscopic analysis. Neighboring samples from the same livers were snap-frozen in liquid nitrogen, and later used for RNA isolation with Trizol (Invitrogen Life Technologies, Carlsbad, California, USA), as described previously [9]. Quantification of RNA was performed with a spectrophotometer, with purity and integrity of RNA verified by 260/280 ratios and by agarose gel electrophoresis.

Total RNA samples were treated with RNAse-free DNAse I (Invitrogen Life Technologies, U.S.A.) to remove any contaminating genomic DNA. Reverse transcription was performed with Superscript II reverse transcriptase and oligo (dT)_12–18 _(Invitrogen) according to the manufacturer's instructions. cDNA pools were subjected to real-time kinetic PCR on a Mx-4000 Multiplex Quantitative PCR (Stratagene, La Jolla, California, USA) using SYBR Green I as a double-strand DNA-specific binding dye to quantify mRNA expression for IL-12, IFN-γ, IFN-α, IFN-β, TNFα, IL-4, IL-5, IL-18, IL-23, IL-27, RRV structural protein VP6, and the nonstructural protein NSP3. PCR amplifications were performed with specific primers outlined in Table 1 in a total volume of 20 μl containing 0.1 pmol of each primer, 10 μl of 2× Brilliant SYBR QPCR master mix (Stratagene), 3 nM of 1:500 diluted reference dye (ROX), 1 μl of 1:5 diluted cDNA and nuclease free water, after initial denaturation at 95°C and 40–45 cycles (95°C for 30 seconds, 55°C for 1 minute and 72°C for 30 seconds). The levels of gene expression were calculated as a ratio to glyceraldehyde-3-phosphate dehydrogenase (GAPDH), as described previously [9].

**Table 1:** Primers used in real-time PCR to quantify levels of mRNA expression.

**Primers**	**Sequences**
IFNγ	Forward: 5'- GGCTGTCCCTGAAAGAAAGC -3' Reverse: 5'- GAGCGAGTTATTTGTCATTCGG -3'
TNFα	Forward: 5'- AAGGGAGAGTGGTCAGGTTGCC -3' Reverse: 5'- CCTCAGGGAAGAGTCTGGAAAGG -3'
IFNα	Forward: 5'- GACTTTGGATTTCCCCTGGAG -3' Reverse: 5'- AAGCCTTTGATGTGAAGAGGTTC -3'
IFNβ	Forward: 5'- GTTACACTGCCTTTGCCATCC -3' Reverse: 5'- CAACAATAGTCTCATTCCACCCAG -3'
IL-4	Forward: 5'- CCACGGATGCGACAAAAATC Reverse: 5'- TGTTCTTCGTTGCTGTGAGGAC
IL-5	Forward: 5'- TCCCTGCTACTCTCCCCAAAC Reverse: 5'- TGGCACAGTCTGATTCATACATAGG
IL-18	Forward: 5'- AAATGGAGACCTGGAATCAGAC -3' Reverse: 5'- TTTGTCAACGAAGAGAACTTGG -3'
IL-23	Forward: 5'-CCCGTATCCAGTGTGAAGATG -3' Reverse: 5'- TGTCAGAGTCAAGCAGGTGC -3'
IL-27	Forward: 5'- TGTTCAAAGGAGGAGGAGGAC -3' Reverse: 5'- GGATGACACCTGATTGGGG -3'
Rotavirus VP6	Forward: 5'- GCGGTAGCGGTGTTATTTCC -3' Reverse: 5'- TTGTTTTGCTTGCGTCGG -3'
Rotavirus NSP3	Forward: 5'- TGTCAAGAGAATACCTGGGAAATC -3' Reverse: 5'- GGAATCATCAACTTCAACTTCACC -3'
GAPDH	Forward: 5'- TGGTTTGACAATGAATACGGCTAC Reverse: 5'- GGTGGGTGGTCCAAGGTTTC

### Isolation and fluorometric analysis of liver cells

Single cell suspensions of freshly harvested livers were obtained from mice 14 days after RRV or saline injection by mincing the livers, followed by passing through a nylon mesh. Mononuclear cells were suspended in RPMI Medium 1640 (Invitrogen, USA) and then centrifuged at 270 *g *for 10 minutes at 4°C. After lysis of the erythrocytes with RBC lysis buffer (0.15 M NH_4_Cl, 10 mM KHCO_3_, and 0.1 mM Na_2_EDTA, pH 7.2), cells were washed with PBS supplemented with 3% fetal calf serum and counted using a Neubauer chamber. Cells were stained by incubation with the following antibodies: anti-mouse CD3-FITC, anti-mouse CD4-PE, anti-mouse CD8a-PE, and anti-mouse neutrophil (CD11b-FITC and GR1-PE) (BD Biosciences, San Jose, California, USA). Then, two-color fluorometric analyses were performed in FACS buffer (PBS containing 0.01% sodium azide and 2% {v/v} FCS) as described previously [9]. Cells were then analyzed using a FACSCalibur dual-laser flow cytometer (BD Biosciences), with excitation at 488 and 633 nm. At least 150,000 events were acquired on the FACSCalibur for each sample, with non-viable cells and cellular debris excluded by forward and side scatter gating. Data were analyzed using CellQuest software (BD Biosciences), as described previously [9].

### Statistical analysis

Values are expressed as mean ± SD, and statistical significance was determined by unpaired t test between IL-12KO mice injected with RRV or saline (controls) unless noted otherwise, assuming normal distribution, with a significance level of P < 0.05.

## Results

### Outcome of IL-12KO mice after RRV infection

**Figure 1:**
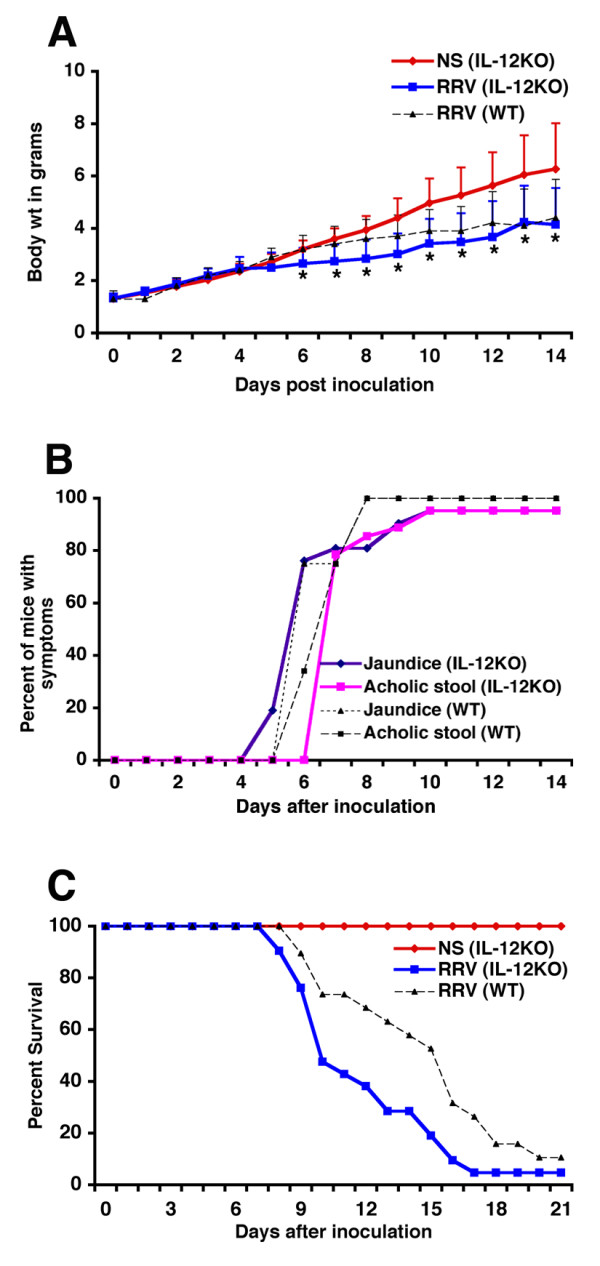
**Outcome of IL-12KO mice infected with RRV. **(A) Injection of RRV into newborn Balb/c mice leads to growth failure, as demonstrated by a significantly lower body weight when compared to non-infected mice (P < 0.05). RRV injection also leads to symptoms of cholestasis in mice between 4–8 days (B), and to decreased survival (C). The poor growth, onset of cholestasis, and decreased survival are similar to wild-type (WT) mice infected with RRV. Total number of animals at the time of injection of RRV or saline: IL-12KO-saline = 8, IL-12KO-RRV = 21, WT-RRV = 19. During the course of the study, all mice injected with normal saline remained healthy. In RRV injected IL-12KO mice, 20 out of 21 animal had died by day 17; similarly, 17 out of 19 RRV infected WT mice had died by day 21.

Administration of 1.5 × 10^6 ^ffu of RRV intraperitoneally into WT mice in the first 24 hours of life resulted in the onset of jaundice and acholic stools within 1 week, with impaired growth, and <10% survival beyond 3 weeks, as described previously [9]. IL-12KO mice did not display overt abnormalities after saline administration, grew well during the suckling period and survived into adulthood. In contrast, IL-12KO mice injected with RRV developed jaundice and acholic stools within 1 week, had poor growth, and only 10% survived beyond 3 weeks of life in a fashion similar to the outcome of WT mice challenged with RRV (Figure 1A–C). Collectively, these data demonstrated that loss of IL-12 did not protect neonatal mice from the development of progressive jaundice after RRV infection.

### Neutrophilic cholangiopathy in IL-12KO mice

**Figure 2:**
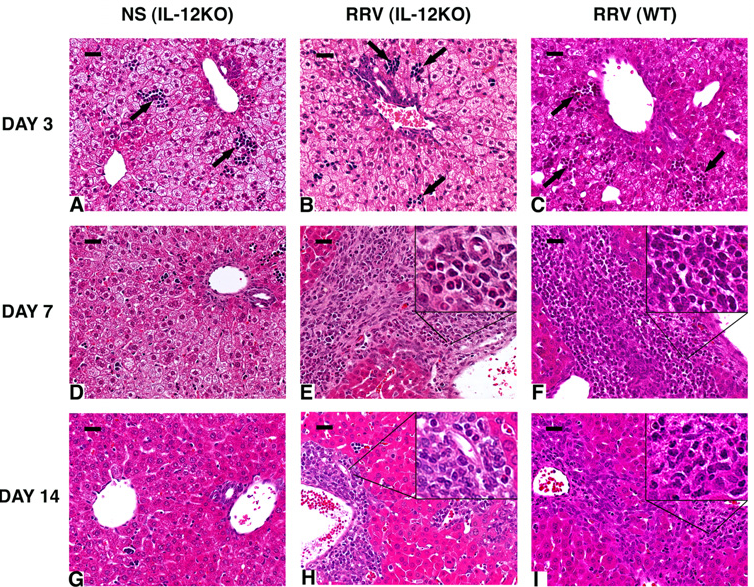
**RRV infection induces biliary inflammation in livers of IL-12KO mice. **Infection of IL-12KO mice with RRV leads to minimal portal changes when compared to saline-injected mice and RRV-infected wild-type (WT) within 3 days (panels A, B, C). In contrast, RRV induces a remarkable portal expansion at 7 and 14 days in both IL-12KO and WT mice (panels E, F, H, I). Although the portal expansion has the same extent in both genotypes, IL12-KO livers have a mixed cellular infiltration with a predominance of neutrophils, while the portal spaces of WT livers contain primarily lymphocytes (insets in panels E, F, H, I). Arrows point to extramedullary hematopoiesis at day 3; N = 15–20 mice in each group; scale bar (left upper portion of each panel): 100 μ.

**Figure 3:**
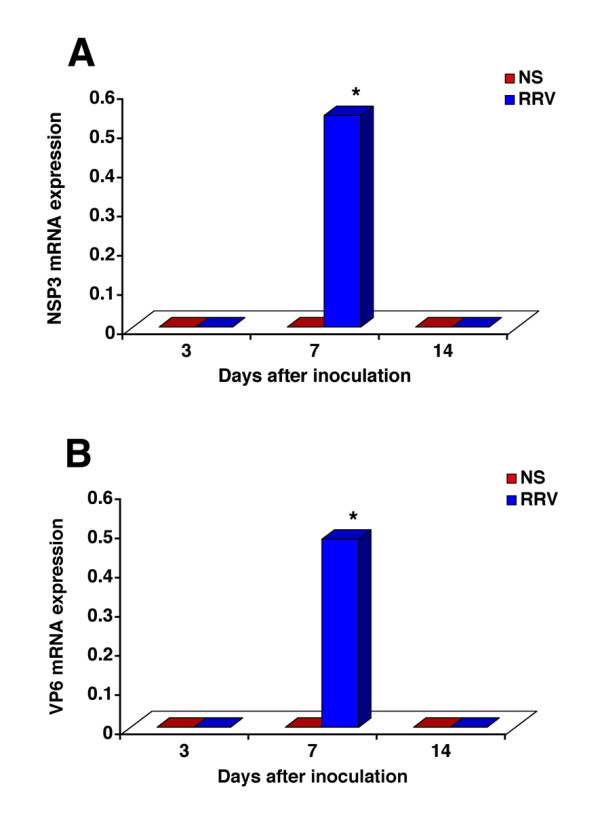
**Clearance of mRNA expression for viral proteins. **The hepatic expression of mRNA encoding RRV nonstructural (panel A: NSP3) and structural (panel B: VP6) proteins in IL-12KO mice was undetectable at days 3, high at day 7, and undetectable at day 14 after infection. P < 0.05; N = 4–6 mice per group at each time point.

To determine the cellular basis of progressive jaundice in IL-12KO mice, we examined histological sections of livers at 3, 7, and 14 days after saline or RRV administration. Livers of IL-12KO mice injected with saline had normal lobular architecture and residual extramedullary hematopoiesis that is typically seen in the first few days of life (Figure 2). Following RRV, however, IL-12KO mice displayed a mild periductal inflammation at 3 days, which progressed to portal expansion by inflammatory cells and duct profiles at 7 and 14 days. The inflammatory pattern at 3 days was similar to that observed in livers of WT mice injected with RRV (Figure 2). However, the cellular components differed at days 7 and 14, with a mixed inflammatory infiltrate containing predominantly neutrophils in IL-12KO mice, while lymphocytes were the predominant cell types in WT mice (Figure 2). To precisely determine the extent to which neutrophils infiltrate the portal space of IL-12KO mice, we quantified T lymphocytes and neutrophils by flow cytometry at 14 days after RRV challenge. We found that the total number of hepatic lymphocytes and neutrophils increased in IL-12KO mice infected with RRV 2–4-fold above saline controls (Table 2). Interestingly, while the increase of CD3/CD4+ and CD3/CD8+ cells was similar to WT mice infected with RRV, the number of neutrophils (dual staining by CD11b-FITC and GR1-PE) in IL-12KO livers was greater than in WT mice after RRV. The changes in the nature of the inflammatory response induced by the loss of IL-12 did not impair the ability of the mice to effectively clear RRV, as demonstrated by the inability to detect the mRNA for structural (VP6) and nonstructural (NSP3) proteins by PCR 14 days after RRV challenge (Figure 3).

**Table 2:** Number (× 10^6 ^cells) of hepatic mononuclear cells 14 days after RRV challenge

**Genotype**	**Injection**	**CD3/CD4+**	**CD3/CD8+**	**Neutrophils**
IL-12KO	Saline	0.26 ± 0.1	0.14 ± 0.03	0.39 ± 0.2
IL-12KO	RRV	0.56 ± 0.1*	0.50 ± 0.2*	1.80 ± 0.01^*@^
Wild-type	RRV	0.70 ± 0.03*	0.56 ± 0.2*	1.0 ± 0.2*

*P < 0.01 when compared to IL-12KO mice treated with saline.

^@^P < 0.05 when IL-12KO mice treated with RRV are compared to wild-type mice also treated with RRV.

**Figure 4:**
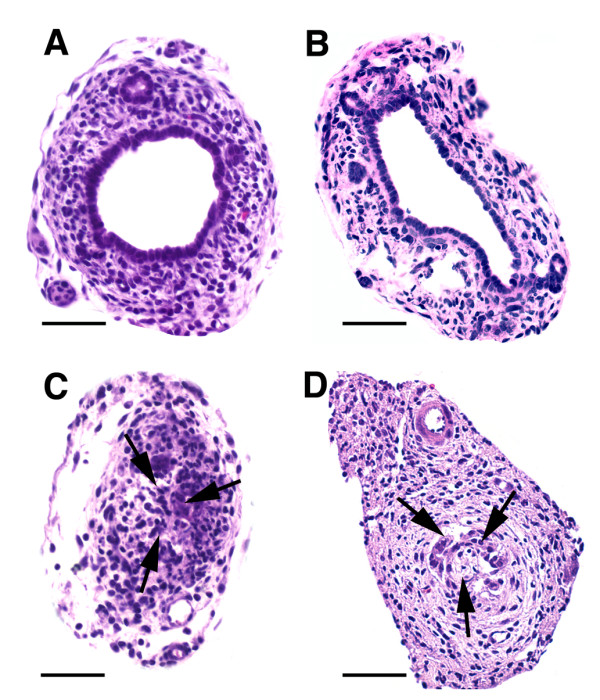
**Representative cross sections of extrahepatic bile ducts in IL-12KO mice. **Panels A and B show the normal epithelium and unobstructed lumen at 7 and 14 days after the injection of saline in the first 24 hours of life. In contrast, the injection of RRV leads to an obstruction of the lumen by inflammatory cells at day 7, and by sloughed epithelial cells of cellular debris at 14 days (panels C, D). Scale bar: 100 μ.

**Figure 5:**
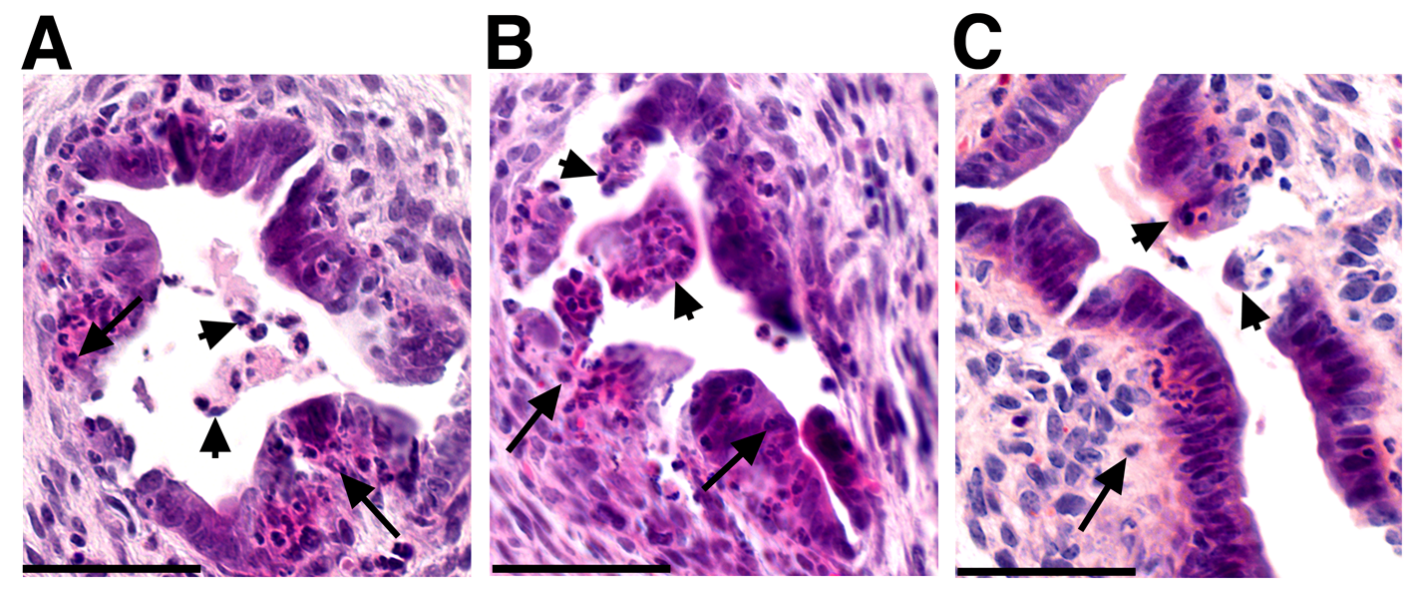
**Neutrophil-rich biliary infiltration in IL-12KO mice. **Representative cross sections of extrahepatic bile ducts in IL-12KO mice 14 days after infection with RRV soon after birth show exfoliation and sloughing of the epithelium (short arrows), associated with an epithelial infiltration by neutrophils (arrows). Scale bar: 100 μ.

To directly determine whether RRV induced cholangiopathy of the extrahepatic biliary tree in IL-12KO mice, we analyzed serial sections of extrahepatic bile ducts 7 and 14 days after RRV challenge. These time points were chosen because they have been reported to display a typical inflammatory obstruction following RRV infection (at 7 days), followed by matrix components and minimal inflammatory cells in atretic segments of bile ducts (at 14 days). We found obstruction of extrahepatic bile ducts in IL-12KO mice (Figure 4). The segments of obstruction, however, displayed ongoing epithelial injury and loss of epithelial integrity, with areas of exfoliation and invasion of the layer of cholangiocytes by neutrophils (Figure 5). Altogether, these data suggest that the in vivo loss of IL-12 did not impair viral clearance nor modify the long-term outcome of RRV-induced biliary atresia. However, the predominant neutrophilic infiltration suggested that neutrophils, rather than lymphocytes, may be the cellular effectors of bile duct obstruction in IL-12KO mice.

### Predominant expression of interferons and TNFα in IL-12KO mice

**Figure 6:**
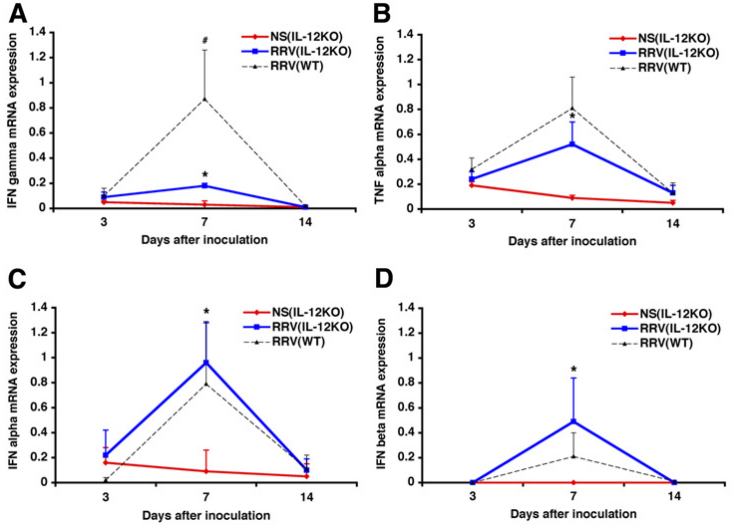
**Expression of proinflammatory cytokines in IL-12KO mice. **RRV infection in the first day of life leads to an increased hepatic mRNA expression of IFNγ (A), TNFα (B), IFNα (C) and IFNβ (D) in IL-12KO mice above levels of saline-injected IL-12KO mice and WT mice injected with RRV. Also shown are the levels of expression. Levels of expression are shown as a ratio to GAPDH; *P < 0.05 for IL-12KO mice injected with RRV vs saline; #P < 0.05 for IL-12KO mice are compared to WT, both after RRV infection; N = 4–6 mice per group at each time point.

**Figure 7:**
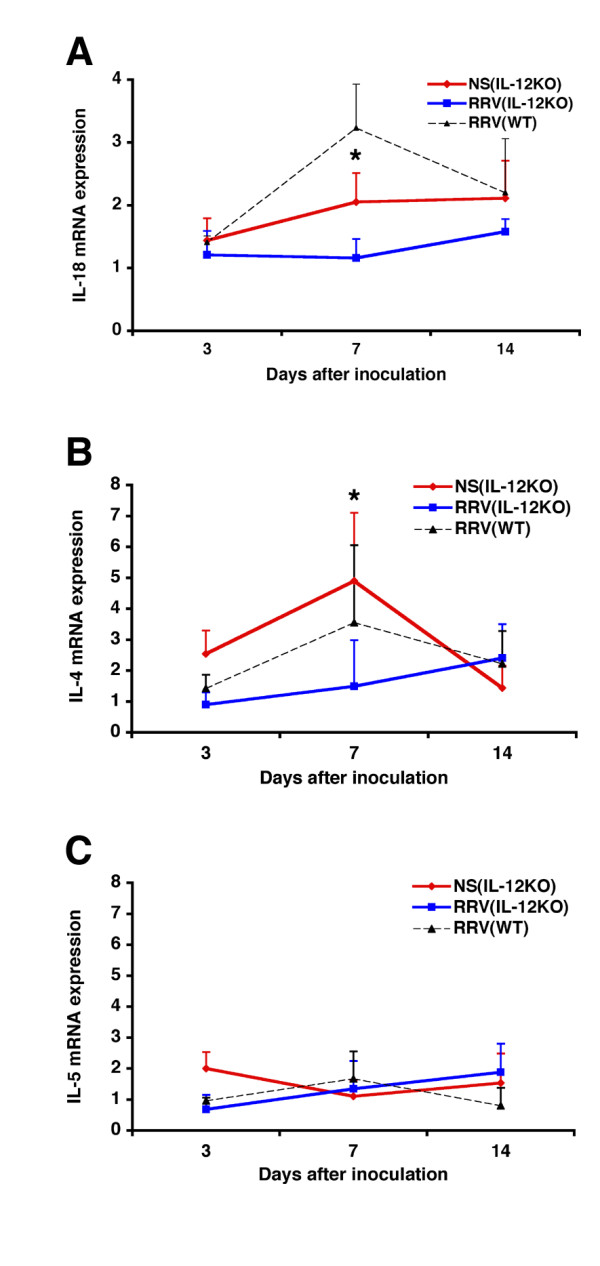
**Expression of IL-18 and Th2 cytokines in IL-12KO mice. **RRV infection in the first day of life leads to a decrease in the hepatic mRNA expression of IL-18 (A) and IL-4 (B), with no changes in IL-5 mRNA (C). Levels of expression are shown as a ratio to GAPDH; *P < 0.05; N = 4–6 mice per group at each time point.

To explore the potential mechanisms regulating the neutrophil-rich cholangiopathy and duct obstruction in IL-12KO mice, we determined the hepatic mRNA expression of the proinflammatory cytokines IFNγ and TNFα. Levels of mRNA expression for both cytokines increased in RRV-injected IL-12KO mice above controls (Figure 6A, B). Interestingly, a comparison of the mRNA levels for IFNγ following RRV challenge between IL-12KO and WT mice demonstrated that the rise of IFNγ was higher in WT mice. These findings suggest that the loss of IL-12 reduced but did not abolish the development of a Th1-like phenotype in the hepatic environment. Based on a previous report showing the role of type 1 interferons in generating a Th1 response in IL-12KO mice [12], we determined the expression of IFNα and IFNβ. We found that the hepatic levels of mRNA expression for both cytokines in RRV-injected IL-12KO mice increased significantly above levels of saline-injected controls, with a peak expression at 7 days that was similar to levels observed in RRV-infected WT mice (Figure 6C, D). In contrast, the levels of mRNA expression for the Th1 inducer cytokines IL-18 and the IL-12 related cytokines IL-23 and IL-27 were either decreased (for IL-18; Figure 7) or undetectable (for IL-23 and IL27; data not shown) after RRV infection. The low levels of expression for IL-18 support a lack of potential involvement of IL-18 in the development of the Th1-like phenotype in IL-12KO mice. Interestingly, the hepatic expression of prototype Th2 cytokines either decreased (IL-4; P < 0.05) or did not change (IL-5) in IL-12KO mice after RRV infection (Figure 7). This is consistent with a dominant activation of proinflammatory cytokines in the hepatic environment during the course of biliary injury and obstruction.

## Discussion

Despite the pivotal role of IL-12 as an innate cytokine and a promoter of the Th1 response, the genetic loss of functional IL-12 did not protect young mice from the development of biliary obstruction after RRV challenge. Following neonatal viral infection, IL-12-deficient mice developed jaundice and acholic stools by 7 days of life, a time when the liver underwent a remarkable expansion of inflammatory infiltrate in the portal space. Although high levels of structural and non-structural viral elements were detected at this phase of injury, loss of IL-12 did not impair viral clearance at later phases. The portal inflammation was of mixed cellularity, with a remarkable presence of neutrophils at 7 and 14 days, which is in contrast to the predominantly lymphocytic infiltrate typically observed in livers of wild-type mice at the same time points after RRV infection. Notably, neutrophils also infiltrated the extrahepatic bile ducts, where they formed subepithelial clusters or invaded the epithelium. These changes were clearly associated with lumenal obstruction and interruption of bile flow. While the loss of IL-12 did not modify the final outcome of the inflammatory target and obstruction of extrahepatic bile ducts, analysis of IL-12-deficient livers identified two biological components with potential roles in the regulation of neonatal cholangiopathy: 1) neutrophils as cellular effectors of duct injury, and 2) type-1 interferons as an alternate molecular circuit to induce a proinflammatory microenvironment.

Neutrophils are key components of the initial inflammatory response to tissue injury. In previous studies of experimental biliary atresia, neutrophils accumulated near intrahepatic bile ducts in early phases of liver injury after RRV challenge, but became inconspicuous as the portal inflammation expanded by the accumulation of lymphocytes, and bile ducts underwent obstruction by 7 days [9]. In the current study, we found that this temporal switch to a lymphocyte-rich population in the portal spaces is controlled, at least in part, by IL-12, as demonstrated by the contribution of neutrophils to the expansion of the portal inflammation at 7 and 14 days (time of bile duct obstruction), as well as by their presence near and within injured epithelium of extrahepatic bile ducts. These findings are of particular interest in view of the previous report of an increase in the number of neutrophils when mice lacking IFNγ were subjected to the same model of RRV-induced biliary atresia. Without IFNγ, the number of hepatic neutrophils increased by 62% above non-transgenic littermates 7 days after RRV challenge [9]. However, this increase was only transient and was not sufficient to promote obstruction of bile ducts owing to a generalized attenuation of the lymphocytic infiltration of extrahepatic bile ducts in mice lacking IFNγ.

The differences in the outcome of biliary obstruction between mice lacking IFNγ and IL-12 are probably due, at least in part, to the combined effects of the neutrophilic infiltration in IL-12 deficient mice through day 14 after RRV challenge and the mild-moderate increase in the expression of IFNγ. Although lower than the expression in WT mice, the rise in IFNγ in IL-12-deficient mice was sufficient to produce a Th1 pro-inflammatory response. Collectively, these findings suggest that young mice activate accessory molecular networks that trigger an increase in IFNγ and maintain a neutrophil-rich mixed inflammatory infiltration in the hepatobiliary system in the absence of IL-12.

The production of IL-12 by phagocytic mononuclear cells and dendritic cells is a key component of the molecular networks that induce functional commitment of naïve CD4 T lymphocytes to a Th1 proinflammatory phenotype [13-15]. In keeping with this physiologic role, mRNA expression of IL-12 increases remarkably after RRV infection [9]. Here, we show that such an increase is not essential for the expression of the proinflammatory response in experimental biliary atresia, as demonstrated by the increases in mRNA levels for IFNγ, and TNFα at the onset of jaundice and acholic stools in mice lacking IL-12. The increase in IFNγ mRNA, however, was of lower magnitude when compared to the levels observed in wild-type mice challenged with RRV. Although of lower magnitude, this increase may have been sufficient to promote the obstruction of bile ducts in IL-12KO mice. Although IL-18, -23, and -27 are candidate cytokines able to trigger the expression of IFNγ [16-19], we found no increase in their levels of mRNA expression in livers of IL-12 deficient mice. Interestingly, the lack of expression of IL-23 and -27 are in accordance with the requirement of p40 as a critical subunit to form functional heterodimers with p19 (to generate IL-23) and p28 (for IL-27; p40 shares high homology to the EB12 subunit). Thus, the loss of IL-23 and -27 induced by the deficiency of p40 disrupts the network among these related cytokines. Despite this disruption, the injury and obstruction of extrahepatic bile ducts proceeded in a timely fashion, albeit with different cellular components. This may have been facilitated by type I interferons, as supported by the increase in the mRNA levels for IFNα and IFNβ in IL-12-deficient mice. Collectively, these data are consistent with a biological setting in which type-1 interferons work independently of IL-12 to induce the expression of IFNγ and other Th1 proinflammatory cytokines after RRV challenge. A similar biological setting was previously demonstrated in IL-12-deficient mice subjected to lymphocytic choriomeningitis virus infection [20]. Direct proof that an IFNα/β-dependent circuit regulates induction of IFNγ and duct obstruction in IL-12KO mice will require the analysis of the biliary phenotype following the in vivo inactivation of type-1 interferons in the same experimental model.

## Conclusion

The genetic loss of functional IL-12 in vivo does not protect neonatal mice from an inflammatory-driven injury and obstruction of extrahepatic bile ducts after a viral insult. However, the inflammatory response displayed a persistent neutrophilic response and a gradual rise in the expression of type-1 interferons at the time of duct obstruction. Exogenous administration of IFNα before or at the time of RRV challenge was previously reported to significantly alleviate the progression of jaundice and cholestasis in this mouse model of bile duct obstruction [21]. In these experiments, however, the authors did not examine the impact of RRV challenge or administration of IFNα on the native production of type-1 or -2 interferons, or on other molecules that may regulate biliary injury and obstruction. In this context, the findings in IL-12KO mice presented herein further underscore the importance of future studies to address the independent or synergistic roles of type-1 interferons and neutrophils in the development of experimental biliary atresia in mice.

## Abbreviations

RRV: *Rhesus *rotavirus

IL: Interleukin

IL-12KO: Interleukin-12 knockout

WT: Wild type

IFNγ : Interferon gamma

IFNα : Interferon alpha

IFNβ : Interferon beta

mRNA: Messenger RNA

TNFα : Tumor necrosis factor alpha

## Competing interests

The author(s) declare that they have no competing interests.

## Authors' contributions

SKM: Study design, carried out the majority of experiments, analyzed data, drafting of the manuscript.

PKS: Animal phenotyping, flow cytometric analysis, data analysis.

GS: Animal phenotyping, histopathology, data analysis.

JAB: Design and supervision of the study, data analysis, drafting of the manuscript.

## Pre-publication history

The pre-publication history for this paper can be accessed here:


